# Influence of a Foot Insole for a Down Syndrome Patient with a Flat Foot: A Case Study

**DOI:** 10.3390/medicina56050219

**Published:** 2020-05-06

**Authors:** Yusuke Endo, Yoshihide Kanai, Arito Yozu, Yasuto Kobayashi, Takashi Fukaya, Hirotaka Mutsuzaki

**Affiliations:** 1 Department of Physical Therapy, Faculty of Health Science, Health Science University, 7187 Kodachi, Fujikawaguchiko-machi, Minamitsuru-gun, Yamanashi 401-0380, Japan; 2 Department of Physical Therapy, Saitama Medical University, 981 Kawakado, Moroyama-machi, Iruma-gun, Saitama 350-0496, Japan; kanai-ptd@umin.ac.jp; 3 Center for Medical Sciences, Ibaraki Prefectural University of Health Sciences, 4669-2 Ami, Inashiki-gun, Ibaraki 300-0394, Japan; yodu-jscn@umin.net (A.Y.); mutsuzaki@ipu.ac.jp (H.M.); 4 Department of Rehabilitation Medicine, Ibaraki Prefectural University of Health Sciences Hospital, 4733 Ami, Inashiki-gun, Ibaraki 300-0331, Japan; 5 Department of Sports Management, Faculty of Business and Public Administration, Sakushin Gakuin University, 908 Takeshita-machi, Utsunomiya-shi, Tochigi 321-3295, Japan; kyasuto@sakushin-u.ac.jp; 6 Department of Physical Therapy, Tsukuba International University, 6-8-33, Manabe, Tsuchiura-shi, Ibaraki 300-0051, Japan; t-fukaya@tius.ac.jp; 7 Department of Orthopedic Surgery, Ibaraki Prefectural University of Health Sciences Hospital, 4733 Ami, Inashiki-gun, Ibaraki 300-0331, Japan

**Keywords:** down syndrome, flat foot, gait analysis, knee joint kinematics, insole

## Abstract

*Background and Objectives*: Patients with Down syndrome have many orthopedic problems including flat foot. Insertion of an insole for a flat foot provides support to the medial longitudinal arch; thus, insole therapy is often used to treat a flat foot. However, the influence of an insole insertion on the knee joint kinematics for a patient with Down syndrome is unknown. This study aimed to elucidate the influence of an insole for a flat foot on the knee kinematics during gait for a patient with Down syndrome. *Materials and Methods*: The subject was a 22-year-old male with Down syndrome who had a flat foot. The knee joint angle during the gait was measured using a 3D motion capture system that consisted of eight infrared cameras. *Results*: The gait analysis demonstrated a reduction in the knee flexion angle during double knee action. The knee valgus and tibial internal rotation angles also decreased during the loading response phase while wearing shoes that contained the insole. *Conclusions*: As the angle of the knee joint decreased during the gait, it was considered that the stability of the knee joint improved by inserting the insole. In particular, there was a large difference in the tibial internal rotation angle when the insole was inserted. It is thus hypothesized that the insole contributes to the rotational stability of the knee joint. This study suggests that knee stability may improve and that gait becomes more stable when a Down syndrome patient with a flat foot wears an insole.

## 1. Introduction

A large number of patients with Down syndrome have problems associated with their musculoskeletal system and joints. Foot and ankle deformities are particularly recognized as common orthopedic problems in patients with Down syndrome. Flat foot is also a common orthopedic complaint for patients with Down syndrome [1]. A previous radiographic study demonstrated that 58% of patients with Down syndrome have flat foot [2]. It is well known that the foot arch supports the body weight and decentralizes plantar pressure. The general treatment for flat foot is the use of insoles, which is an important part of physical therapy. With regard to the effect of insole insertion, there is a report that indicates that insole insertion significantly reduced the ankle eversion angle [3]. The ankle and knee joints are articulated and it is expected that the kinematics of the ankle joint will affect the knee joint. However, there are reports that static foot structure does not affect knee gait kinematics [4], though the effect on knee kinematics is unknown. Excessive motion of the knee joint may increase the mechanical load on the knee joint and lead to the development of knee osteoarthritis.

Previous studies have also recommended that patients with Down syndrome should use an insole [5]. In addition to bone abnormalities and deformities, obesity is also associated with Down syndrome. These physical problems can cumulatively result in a decline in gait function. The incidence of flat foot for patients with Down syndrome is estimated to be 27.0–58.0% [2,6]; 88.9% of these patients are treated with insole insertions [6]. It is known that inserting an insole as a treatment method for a flat foot is important and commonly used. Previous studies have reported that patients with Down syndrome exhibit a specific gait. For example, Down syndrome patients have external rotation of the hip joint, flexion and valgus of the knee joints, and external rotation of the tibia [7]. Insole insertion is widely prescribed in patients with Down syndrome for static foot structure problems such as flat foot, which may result in improved dynamic stability of the knee joint and act to protect the knee joint. However, the effect of insole insertion on the gait kinematics of the knee joint is unknown. The purpose of this study was thus to elucidate whether an insole affects gait kinematics for patients with Down syndrome that have a flat foot. This study examined a patient with Down syndrome who had been prescribed a foot insole.

## 2. Materials and Methods

A 22-year-old male (height = 152 cm, weight = 58.5 kg) patient with Down syndrome participated in this study. He was diagnosed with bilateral flat foot two years ago, and the calcaneal angle was 13.9° for his right barefoot and 15.1° for his left barefoot. The normal range of the calcaneal pitch is reported to be 18 to 20° in the literature [8]; however, other studies have reported that 17 to 32° is normal [9]. Calcaneal angles below this range are consistent with a flat foot. The patient was prescribed a foot insole for medial longitudinal arch support. The patient could walk without assistance and devices and though he demonstrated a mild intellectual impairment, communication was possible. The kinematic experiment was carried out using an insole placed in the patient’s shoes.

**Figure 1:**
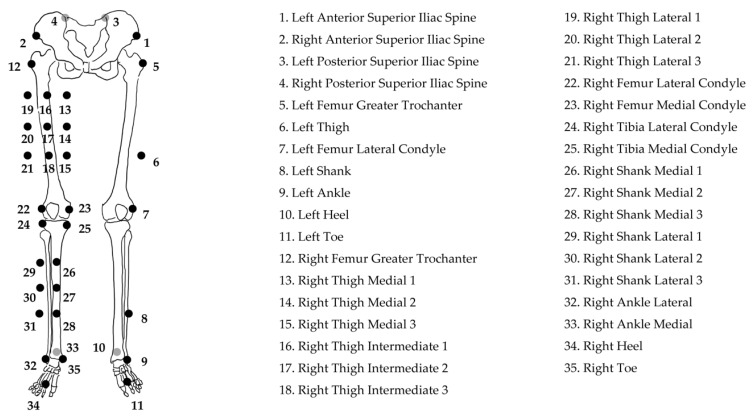
The reflective marker set for the point cluster technique.

The gait analysis was performed under two conditions: insole-inserted shoes and non-inserted shoes. The knee joint angle data of the dominant lower extremity was measured for the gait analysis. Kinematic data during gait was acquired using a motion capture system (Vicon Nexus, Oxford Metrix, Oxford, UK), which consisted of eight infrared reflective cameras with a sampling rate of 100 Hz. Thirty-five 14 mm infrared reflective markers were placed on the patient’s lower extremities (Figure 1) and the point cluster technique [10,11] was used as a reflective marker for the set model to calculate the knee joint angle during the gait. The point cluster technique was developed to assess the tibial rotation angle accurately. Referring to the previous study [12], the point cluster method was calculated as follows. Using the static standing posture as the reference posture and the transformation matrix in the reference posture, the coordinate system of the segment at each gait time was obtained from the principal axis of the segment in motion. Next, the rotation matrix R(t)k between the thigh and shank segments, which is converted from the rotation matrix R(t)_th_ of the thigh segment coordinate axis to the rotation matrix R(t)_sh_ of the shank segment coordinate axis, was calculated from the following equation to obtain the knee joint angle.

$$R\left(t\right)k=R{\left(t\right)}_{\mathrm{sh}}R{\left(t\right)}_{\mathrm{th}}^{-1}$$

The knee joint angle was calculated using MATLAB R2016b (MathWorks Inc., Natick, MA, USA). The obtained knee joint angle was normalized while considering one gait cycle as heel contact to heel contact for the ipsilateral lower extremity. The knee joint data for three gait cycles were averaged and compared for the conditions with and without the insole. The patient was instructed to walk at his usual preferred speed. The measurements were performed for the patient walking without the insole first, followed by walking with the insole. 

This study was approved by the ethics committee of the Ibaraki Prefectural University of Health Sciences on October 10, 2017 (approval No. 778), and written informed consent was obtained from the subject and his family.

## 3. Results

**Figure 2:**
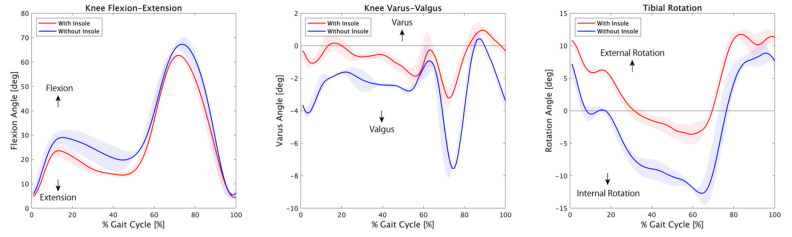
The knee joint angle from the dominant leg during the gait cycle.

The mean angles of the dominant knee joint were calculated from three gait trials and are plotted in Figure 2 and Table 1. The red-filled area around the solid line represents the standard deviation in the insole conditions and the blue-filled area represents the standard deviation in the without insole conditions.

The mean angle and standard deviation of knee joint flexion was 26.53 ± 1.95° with the insole and 31.73 ± 3.45° without the insole. The knee flexion angle decreased when the patient wore shoes with the insole in comparison to wearing shoes without the insole. The knee flexion angle during the double knee action decreased. The maximum knee flexion angles for the first peak during double knee action with and without the insole were 23.60° and 29.03°, respectively. The maximum knee flexion angles for the second peak during double knee action with and without the insole were 62.75° and 67.27°, respectively.

The mean angle and standard deviation of knee joint varus was −0.69 ± 0.45° with the insole and −2.52 ± 0.36° without the insole. The knee valgus angle decreased when the patient wore the shoes with the insole. During the loading response phase, the maximum valgus angle difference was observed between the shoes with the insole (3.21°) and the shoes without the insole (7.55°).

The mean angle and standard deviation of tibial external rotation was 3.78 ± 0.90° with the insole and −2.77 ± 1.41° without the insole. The maximum tibial internal rotation angle for the shoes without the insole (3.65°) was larger than for the shoes with an insole (12.39°) by 60% for a gait cycle that corresponded to the terminal stance phase to the initial swing phase. The gait cycle durations under with insole and without insole conditions were 1.12 ± 0.01 s and 1.27 ± 0.05 s, respectively.

## 4. Discussion

In this study, the knee joint flexion angle decreased when an insole was inserted. This result was observed during double knee action. Double knee action is defined as a biphasic knee joint flexion–extension pattern during a gait cycle [13], which functions as a shock absorption mechanism. By having enough assistance from the insole to help with shock absorption, the knee joint’s excessive flexion angle was reduced in this study.

The tibial internal rotation angle during the gait decreased by inserting the insole; however, the external tibial rotation angle increased. The rotational stability of the knee joint increased since the movement range of the tibial rotation angle was reduced. The increased rotational stability was caused by the effect of the insole on reducing the pronation of the foot. An increase in knee rotational stability is important for joint protection and may prevent osteoarthritis in the long term.

Conversely, the knee joint valgus angle during the gait decreased by inserting the insole. The valgus angle of the knee joint decreased by inserting the insole, which supports the medial longitudinal arch of the foot. This result supported the original hypothesis. In terms of the results for this case study, individual differences are considered to have an influence.

In a previous study that verified the insertion effect of a biplanar insole, it has been reported that the ankle joint eversion angle decreased significantly [3]. However, to our knowledge, there is no report that clarifies the effect of insole insertion on the gait kinematics of patients with Down syndrome. 

With regard to other gait parameters, the gait cycle duration was higher with the insole than that without the insole. Notably, the patient walks with the insole in his daily life, which may have caused the effect of habituation in the gait pattern.

This study has some limitations. The main limitation of this research is that this investigation was performed for a single patient. It is difficult to apply the results obtained in this study to all patients with Down syndrome. We analyzed the dominant lower extremity in order to unify the lower extremities measured, assuming that we would have data of a larger sample size in the future. The foot deformation varies from patient to patient, and accordingly, the insole should be customized to the individual’s foot shape. Further research is warranted to verify the effects of different classes of foot deformations and insole shapes. Long-term follow-up is also necessary to verify whether insole insertion is effective in preventing osteoarthritis.

## 5. Conclusions

This study investigated the effect of insole insertion on knee joint kinematics for a patient with Down syndrome that has a flat foot. By decreasing the angle of the knee joint during the gait, the stability of the knee joint improved upon insertion of the insole. In particular, there was a large difference in the tibial internal rotation angle when the insole was inserted in comparison to the case without the insole. It is hypothesized that the insole contributes to the rotational stability of the knee joint. As the knee stability improves, the gait becomes more stable for a patient with Down syndrome that has a flat foot while wearing the insoles. Further examinations of more patients with Down syndrome that have a flat foot are needed to fully establish the effect of insole insertion.

## Figures and Tables

**Table 1:** The mean knee joint angles and standard deviations during gait.

	With Insole		Without Insole	
	Mean	SD	Mean	SD
Knee Flexion-Extension Angle (deg)	26.53	1.95	31.73	3.45
Knee Varus-Valgus Angle (deg)	−0.69	0.45	−2.52	0.36
Tibial Rotation Angle (deg)	3.78	0.90	−2.77	1.41
